# Editorial: Biochemical and endocrinological parameters in animals matrices

**DOI:** 10.3389/fvets.2022.1124569

**Published:** 2023-01-09

**Authors:** Valeria Pasciu, Ana M. Molina-López, Elena Baralla

**Affiliations:** ^1^Department of Veterinary Medicine, University of Sassari, Sassari, Italy; ^2^Department of Anatomy, Comparative Pathological Anatomy and Toxicology, University of Córdoba, Córdoba, Spain

**Keywords:** biochemical parameters, hormonal parameters, animals matrices, nutrition, physiological status

Biochemical and hormonal blood parameters are key factors when evaluating nutrition, sex, age, health, and physiological status (e.g., pregnancy and lactation) of animals (1, 2). Several pathological conditions or contact of the animal with drugs or environmental toxicants, can alter these important parameters. Therefore, their monitoring is fundamental to assess animal's wellbeing. Nevertheless, these procedures can be very challenging, especially when these parameters have to be monitored on wild or protected species (3). In fact, the matrix to analyse, usually blood, is not always easily available without affecting animal welfare or stressing the animal. Thus, alternative matrices, such as fragments of tissues, feces, urine, saliva, milk, or even cellular extracts and culture media can help to face the challenge. For this reason, it is of great importance to flank the traditional blood analysis with alternative matrix use, even validating a method for a specific matrix and species. This Research Topic is aimed at collecting papers suitable to improve our knowledge and understanding on how biochemical and endocrinological parameters and their metabolites, can be monitored in different animal matrices and species. In this way, it is possible to correlate their values with animals' performance, health status and physiological, nutritional, or environmental conditions (4). In addition to this, specific assay methods are collected in order to constitute a useful tool for the scientific community. In this special e-collection there are eight papers covering the above-mentioned aspects.

Data from Pasciu, Nieddu et al. and Pasciu, Sotgiu et al. in their two reported papers, show as in mouflon and in sheep, fecal T3 metabolites (FTMS) can be assayed non-invasively and respecting animal welfare. Authors have analytically and biologically validated a method for FTMs assay in a wild species (mouflon) and in a domestic one (sheep). The biological validation did not show differences in FTM levels between ewes and rams for both mouflon and sheep species. FTM levels showed modifications according to the ambient temperature. In mouflon, higher values were infact observed in both sexes in Spring, when compared to those found in Summer confirming that ambient temperatures is a main driver in T3 fluctuations in wild ungulates. Furthermore, higher values of FTMs were found in lambs when compared to adult sheeps. Moreover, variations were observed in FTMs levels in pregnant sheep, cyclic and early lactation ewes confirming that during lactation, there is an high energetic demand.

The increased energy demand for milk production can pose a risk for hypoglycemia. Data from Atkin et al. show that heat stress pathway proteins were significantly decreased in blood cattle when hypoglycemia was hyperinsulinemic-induced. This confirm that depending on the hypoglycemia degree, the heat shock proteins protective effect may be reduced or even reversed. The monitoring of glucose in cattle's blood is useful to ensure that milk yield is not compromised.

The high energy demand during the peripartum period is investigated by Daudon et al. in dairy cows. They show that some plasma and adipose tissue Fibronectin type III domain-containing proteins (FNDC) may be involved in lipid mobilization and in the regulation of negative energy balance in cattle. In particular they observe an increase of a fragmented product of a FNDC that is probably due to an adaptive response to the high energy demand of lactation in cattle.

The negative energy balance, can be evaluated assaying several biomarkers like β-hydroxybutyrate (BHBA).

BHBA has also been used to indicate subclinical ketosis in dairy cattle (5).

Abdelsattar et al. analyze goats' blood to study the effects of age and dietary BHBA on blood metabolites, immunoglobulins, and hormones (growth hormone and insulin-like growth factor I) in growing goats. They highlight the influence of age on blood composition (especially around weaning) and the beneficial effects of BHBA on the regulation of blood total protein level in young goats.

Theinert et al. generate primary data in healthy cows to serve as reference values for future studies. They use cows' liver and blood. Total lipids (TL: triacylglycerol, TAG; phospholipids, PL; non-esterified fatty acids, NEFA; and cholesterol esters) are measured in liver tissue, while NEFA, BHBA, and cholesterol are analyzed in blood. TL, TAG, NEFA, and cholesterol esters in liver tissue and NEFA in blood increase in the periparturient period. Analyzed parameters vary in relation to age and physiological conditions.

Molle et al. study the impact of morning vs. afternoon grazing on rumen liquor (ammonia and volatile fatty acids) and on blood plasma (Glucose, NEFA, and urea and Insulin, cortisol and ghrelin) parameters, in lactating dairy sheep. The afternoon pasture allocation increased the intake of water-soluble carbohydrates content, influencing rumen fermentation and leading to a rise of glucose and insulin levels in blood. Moreover, the afternoon grazing decreased the level of cortisol and ghrelin, suggesting a higher satiation-relaxing effect. This study opens the door for future research to evaluate the effect of high sugar intakes at pasture on the reproductive efficiency of dairy sheep.

The analysis of biochemical parameters constitutes an useful tool also for other animals products intended for human consumption. According to this, El-Tarabany et al. compare the muscle oxidative stability, carcass traits and meat composition in commercial broilers and spent laying hens. They conclude that using certain precautions, spent hens can be used as meat in order to face the high demand for chicken meat around the world.

In summary, the results of the above mentioned studies represent relevant data describing how Biochemical and Endocrinological parameters determination is fundamental from a one health perspective including animals, human, and environmental welfare.

## Author contributions

All authors listed have made a substantial, direct, and intellectual contribution to the work and approved it for publication.
